# How does a fly die? Insights into ageing from the pathophysiology of *Drosophila* mortality

**DOI:** 10.1007/s11357-024-01158-4

**Published:** 2024-04-20

**Authors:** Eliano dos Santos, Helena M. Cochemé

**Affiliations:** 1 MRC Laboratory of Medical Sciences (LMS), Hammersmith Hospital Campus, Du Cane Road, London, W12 0HS UK; 2 Institute of Clinical Sciences, Hammersmith Hospital Campus, Imperial College London, Du Cane Road, London, W12 0HS UK

**Keywords:** *Drosophila*, Ageing, Lifespan, Pathology, Death

## Abstract

The fruit fly *Drosophila melanogaster* is a common animal model in ageing research. Large populations of flies are used to study the impact of genetic, nutritional and pharmacological interventions on survival. However, the processes through which flies die and their relative prevalence in *Drosophila* populations are still comparatively unknown. Understanding the causes of death in an animal model is essential to dissect the lifespan-extending interventions that are organism- or disease-specific from those broadly applicable to ageing. Here, we review the pathophysiological processes that can lead to fly death and discuss their relation to ageing.

## Introduction

For over a century, the fruit fly *Drosophila melanogaster* (hereafter referred to as *Drosophila* or fly) has served as an important experimental system, at the forefront of many fundamental breakthroughs in genetics and developmental biology [1, 2]. More recently, *Drosophila* has emerged as a valuable model to study metabolic processes in vivo [2, 3].

Due to its short lifespan (approximately 2 months median and 3 months maximum under standard laboratory conditions), *Drosophila* has been widely used in the field of ageing [4]. Lifespan experiments with flies benefit from the ability to have large group sizes and the scope to test essentially limitless numbers of conditions and interventions, compared to mammals which are more restricted from an ethical and financial perspective. Capitalising on strong evolutionary conservation of key metabolic and signalling pathways, interventions that alter longevity were initially discovered in invertebrates such as flies and subsequently shown to be valid in mammals [4].

While death is scored as the outcome in survival assays, we still know surprisingly little about how flies die from old age during experiments. Due to their small body size and rapid desiccation after death, performing autopsies to establish a definitive cause of death is technically challenging. In an attempt to address this gap in our knowledge, here we will highlight some of the physiological processes and organs whose failure has been associated with fly mortality. Our rationale is that insight into the pathophysiology of fly death will improve our overall understanding of the fly model and further strengthen its value for the ageing field.

## Understanding death to study ageing

Organisms raised under ideal environmental conditions still die eventually due to the critical failure of one or more essential organs. This death is preceded by a gradual loss of function and homeostasis over time, which defines the ageing process. Survival has therefore been used as a readout for ageing. However, the following limitations should be taken into account: (1) ideal conditions are an elusive concept; (2) the threshold for critical loss of function to affect survival is organ- and organism-dependent; and (3) individuals within a population succumb to different causes of death.

In humans, treating or preventing a specific disease, as is current clinical practice, can extend median population lifespan given that disease is prevalent in the population and limits survival. Doing so will not necessarily increase disability-free years (or years lived in good health) because ageing leads to the functional decline and disease predisposition of most organs. Targeting survival prolongs life, while targeting ageing prolongs disability-free life or healthspan [5]. To dissect survival from ageing, we must therefore fully understand why model organisms die and apply comprehensive functional scores to fully test life-extending interventions.

In *Drosophila*, death is simply defined by the prolonged absence of movement, excluding conditions that would lead to confounding immobilisation such as higher environmental CO_2_ or hypothermia. Conceptually, death occurs when basic physiological processes that maintain fundamental organ functions cease to work. These causes can affect any organ and can be generally categorised as follows:

1. *Loss of energy.* The inability to produce or use energy can be related to (a) substrate intake, (b) substrate distribution, (c) substrate utilisation, and (d) the regulation of these processes.
2. *Loss of functional units.* Energy-unrelated factors can lead to cell death, including (a) endogenous toxins and (b) exogenous toxins.
3. *Loss of fluid homeostasis.*

Pathophysiological processes that contribute to these causes of death are interlinked and may underly, yet not be the ultimate or immediate cause of death. We have opted to categorise potential causes of death based on functional processes, to enable insights more readily into how different organs can fail with ageing and ultimately cause death, based on survival studies.

**Fig. 1:**
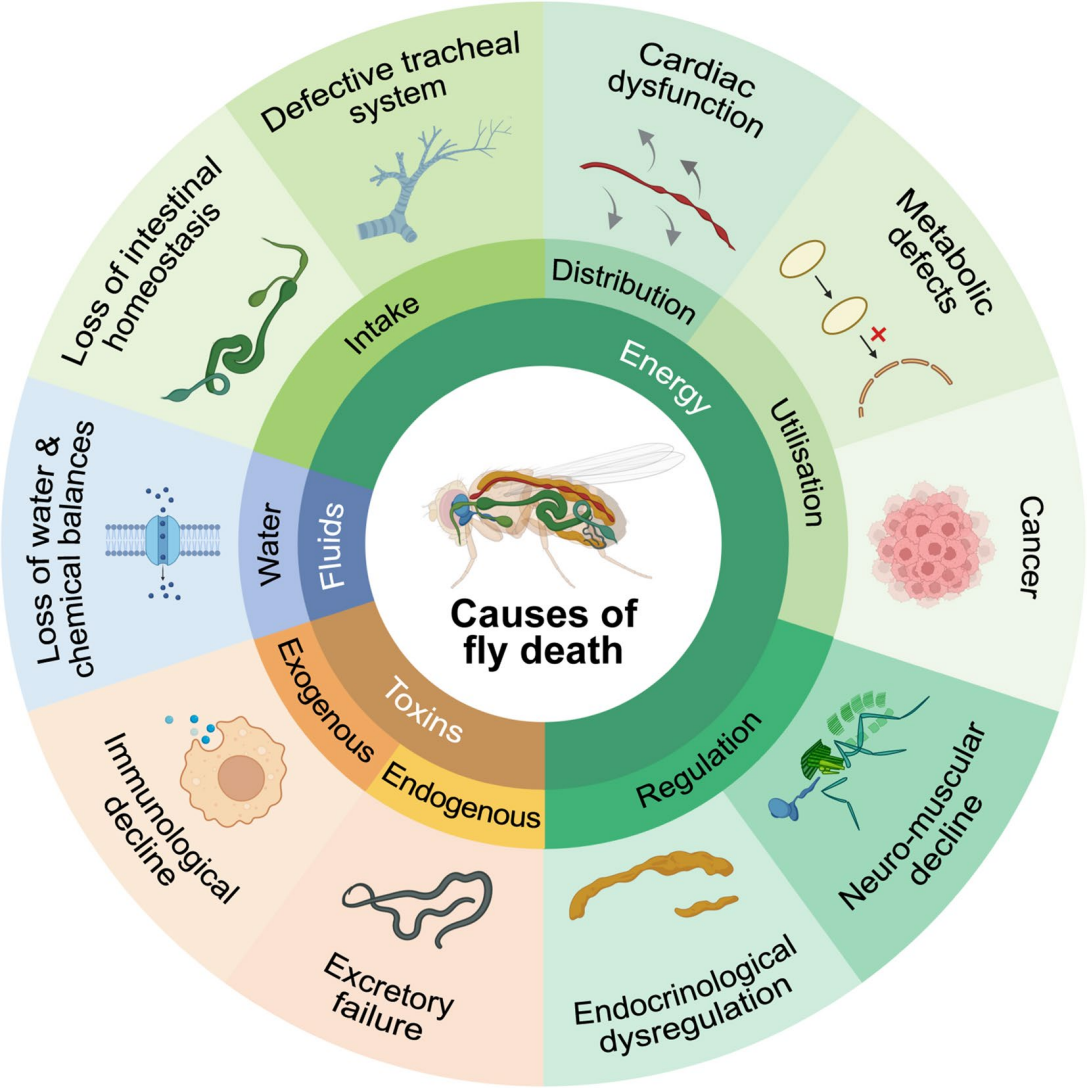
Scheme highlighting a range of physiological systems and organs that undergo age-related decline and are implicated in the pathophysiology of *Drosophila* mortality (created with BioRender.com)

In the following sections, we will therefore discuss how these physiological processes might fail and lead to fly death, taking into account the main organs responsible for their maintenance (Fig. 1).

## Substrate intake: loss of intestinal homeostasis and blocked tracheal system

### Loss of intestinal homeostasis

The fly intestine has been repeatedly shown to alter longevity through multiple genetic and pharmacological interventions affecting its homeostasis, e.g. [6–12]. Aged intestines progressively lose the ability to acidify the copper cell region (a specialised highly acidic compartment within the midgut) [13], which can impact food digestion and allow commensal dysbiosis, further promoted by the decline of innate immunity [7].

Old intestinal epithelium loses its barrier function, which is shown to precede death and correlate with survival [14, 15]. Besides, the epithelium becomes dysplastic following mis-differentiation of aged intestinal stem cells (ISCs) [16, 17]. With age, ISCs overproliferate and are depleted due to an imbalance between the c-Jun N-terminal kinase (JNK) and Notch signalling pathways [16, 17]. Pro-proliferative JNK signalling is upregulated in response to age-related dysbiosis [16, 17]. Anti-proliferative Notch signalling is downregulated due to gene inactivation following age-dependent somatic DNA deletions and large chromosomal rearrangements [18]. Loss of this balance shortens fly lifespan, and only moderate reductions in proliferation increase survival [16], likely through protective aspects such as regeneration and stress resistance [7].

Interestingly, ISCs behave differently depending on their intrinsic sexual identity [19, 20]. Higher levels of proliferation in female ISCs occur as an intestinal adaptation to mating. Females are consequently more resistant to gut stressors than males, but as a downside also more susceptible to dysplasia and tumours [19–21].

Dietary restriction (DR), a classic pro-longevity intervention consisting of decreased nutrient intake without malnutrition, extends survival of females comparatively more than males, which correlates with the differences in gut dysplasia [21]. Indeed, genetic feminisation of the intestine makes male flies shorter-lived but more responsive to DR [21]. The target-of-rapamycin (TOR) pathway inhibitor rapamycin and the Ras pathway inhibitor trametinib also preserve intestinal homeostasis with age and extend fly lifespan with a higher effect size in females [9, 12, 21, 22]. This suggests that loss of intestinal homeostasis may limit fly survival in a sex-dependent manner.

Besides ISCs, but perhaps as a consequence of their mis-differentiation in combination with intrinsic consequences of ageing [7], intestinal somatic cells also functionally decline over time. Indeed, the ability to absorb nutrients is compromised in old flies. Overexpressing a regulator of enterocyte nutrient absorption increases starvation resistance and extends the lifespan of female flies [23]. Altogether, the morphological changes and functional decline of the intestine with age may reach a threshold where substrate intake is sufficiently compromised to initiate systemic failure and death.

### Defective tracheal system

*Drosophila* rely on a network of vessels, called trachea, for delivery and exchange of gasses. Despite the essential role of oxygen in metabolic and redox processes, the tracheal system has mostly been studied in the context of development, and its role in ageing is still poorly understood [24]. Loss of tracheal function can be caused by intrinsic epithelial ageing and loss of morphology or lumen invasion by overproliferation of surrounding tissues, such as in cancer. Furthermore, tracheal branching exhibits plasticity in response to nutritional status, which affects fly physiology—for instance, flies with reduced gut tracheation show increased survival on a deprived diet [25].

Despite being an open network, entry of air to the trachea is regulated by spiracles, valve-like structures important for minimising water loss [26]. Spiracle closure is controlled by muscle contraction, which may deteriorate with age and in turn affect mortality, but this is currently unknown.

## Substrate distribution: cardiac dysfunction and haemolymph

### Cardiac dysfunction

While flies have an open circulatory system, they have a tube-like heart that contracts to induce movement of extracellular fluid, the haemolymph, thereby distributing nutrients, immune cells, hormones, and other signalling molecules throughout the body.

The fly heart suffers multiple changes during ageing, including disrupted diastolic and systolic morphology, impaired speed and function, and arrhythmias [27–31]. This physiological cardiac decline is related to impaired calcium dynamics with age [28, 29, 32], as well as altered gene expression related to myofilaments, calcium handling, proteostasis and response to adrenergic-like signalling [27, 30]. Furthermore, the fly heart becomes stiffer with age [27, 31, 33, 34], associated with the accumulation of specific proteins in the extracellular matrix, resulting in a fibrosis-like phenotype [35, 36]. The age-related decline in heart function seems to be alleviated by challenges that induce physical activity, but without lifespan benefits [37–40].

Some age-related changes in heart morphology, such as decreased diastolic diameter, may in fact be adaptations to systemic ageing. The cytoskeletal protein vinculin has been implicated in this compensatory cardiac remodelling [31]. Heart-specific vinculin overexpression reinforces the cortical cytoskeleton and enhances myofilament organisation, improving cardiac contractility and significantly extending fly lifespan [31].

Age-related cardiac dysfunction is partly recapitulated by feeding flies obesogenic diets with high-fat or high-sugar content [41–44]. These diets shorten fly lifespan, although through heart-independent or indirect mechanisms [45, 46]. In contrast, time-restricted feeding to daylight hours partially protects against diet- and age-induced decline in cardiac function [44] and extends fly lifespan [47].

Besides the heart, changes in haemolymph nutrient carrier proteins can also impact nutrient delivery and regulation. For example, overexpressing the fly orthologue of apolipoprotein D promotes resistance to starvation and extends fly lifespan [48]. Loss of nutrient distribution may be a process that ultimately leads to fly death in a high proportion of a fly population. Further research is needed to understand the impact of maintaining heart function on systemic homeostasis and survival.

## Substrate utilisation: metabolic defects and cancer

### Metabolic defects

The ability to utilise oxygen and nutrients is essential to generate energy. Therefore, disruption of key metabolic pathways in old flies required for the normal functioning of different organs can impact survival. Male fly metabolic rate, inferred from CO_2_ production, was shown to decrease during early adulthood and to stabilise thereafter [49]. In an independent study comparing CO_2_ production with age of different *Drosophila* species, male *D. melanogaster* showed no age difference, and metabolic rates did not correlate with survival across the species [50].

In terms of substrate utilisation, mitochondrial complex I subunits and glycolytic enzymes become less abundant with age, while conversely proteins involved in glutamine-dependent anaplerosis increase [51]. Comparison of metabolomic analysis between 3 and 30 day-old flies reveals an age-dependent metabolic shift from glycolysis to serine metabolism and purine metabolism [52]. While these studies indicate age-related changes in nutrient utilisation, the link to survival remains unclear. Altered nutrient utilisation may instead be an adaptation to systemic ageing or to changes in nutritional demands and chemical balances related to ageing [51].

Multiple studies have shown increased longevity upon genetic or pharmacological interventions in nutrient-sensing and utilisation pathways [4, 53], but whether there are naturally occurring defects in these pathways with ageing critical enough to cause death is unknown.

### Cancer

Cancer can impact survival by affecting the tissue of origin (e.g. gut) or nearby structures (e.g. trachea), as well as by consuming essential nutrients at the expense of neighbouring non-cancerous cells.

Although frequently used as a cancer model due to easily manipulatable genetics, *Drosophila* is mostly composed of post-mitotic cells and thus naturally occurring tumours are relatively rare [54]. Studies focus mainly on the intestine, where tumourigenesis can occur with ageing following ISC dysplasia (see ‘Loss of intestinal homeostasis’). Additionally, ISCs undergo metabolic reprogramming similar to that observed during oncogenic transformation in some cancers [55]. This shift occurs due to age-related decline in mitochondrial calcium, whose transient uptake in young cells increases electron transport chain flux to match energy demand upon proliferation. With lower calcium uptake, old stem cells need to switch to glycolysis for rapid production of ATP, used for hyperplasia [55].

Heterozygous flies for tumour suppressor genes are short-lived in both sexes [56]. Whether the uncontrolled proliferation of cells has a direct impact on survival or indirectly by altering energy utilisation and tissue dysfunction remains to be elucidated.

## Energy regulation: neuronal and endocrinological dysregulation

### Neuro-muscular decline

The major consumers of aerobic energy in the fly are the brain and the flight muscle. At the same time, these tissues are also important regulators of energy processes, from intake and storage, to distribution and utilisation [57].

A fly’s speed and ability to climb decrease with age, hence climbing assays are routinely performed as an indicator of fly healthspan, e.g. [58–61]. This decline can be due to either neuronal or muscular loss-of-function, but most likely a combination of both. Climbing is performed by leg glycolytic muscle, whose ageing is not prevented by DR [61].

Flight function also declines with age [62]. However, young and old flies can tolerate low oxygen conditions to the same extent and can fly at similar speeds [59]. This may be associated with an age-related metabolic shift in these muscles towards glycolysis [63]. Inducing this glycolytic shift at an earlier age shortens fly lifespan [63], suggesting it may be a pathophysiological process preceding fly death.

Besides motility which is also muscle-dependent, neuronal function can be assessed by other functional assays such as feeding behaviour or the analysis of activity and sleep. Flies feed less with age, which reflects lower energetic demands and reproductive outputs [64]. Circadian disruption shortens fly lifespan [65–67], and sleep deteriorates and becomes more fragmented with age [68]. Sleep length was reported to associate with longevity [65–67], although this correlation was challenged by more precise methods of activity monitoring, where chronic sleep deprivation barely impacted on survival [69]. As with other tissues, loss of brain function with age may correlate to a metabolic shift. Brain function depends heavily on glucose supply, which decreases with ageing in the fly head [70]. Restoring glucose levels and improving its uptake by the brain extend fly survival [70].

### Endocrinological decline

Hormones that modulate energy storage and mobilisation can regulate longevity. Haemolymph concentrations of trehalose, the main circulating sugar in *Drosophila*, are regulated by insulin-like peptides (dILPs) and the glucagon-like peptide AKH [3]. Ablation of the cells in the brain responsible for the production of dILPs as well as deletion of specific dILPs extends fly lifespan, despite dysregulation of haemolymph sugar levels [71, 72]. Interestingly, AKH mutant flies also have extended survival [73].

The major steroid hormone characterised in the fly is ecdysone. Mutants deficient for ecdysone synthesis or the ecdysone receptor are long-lived [74, 75]. Although as yet undiscovered, flies are also likely to have an endogenous cortisol-like ligand, since they were found to have a receptor that is responsive to cortisone, which increases their susceptibility to infection, compatible with the known immunosuppressive effect of the drug [76]. Cortisone was shown to extend fly lifespan [77], but more research is needed to understand steroid hormone physiology in the fly and its potential impact on lifespan.

Sex-specific hormone effects also determine survival. For example, juvenile hormone (JH) is responsible for the post-mating shortening of female survival, through overactivation of the innate immune system and its consequent inflammatory response [78–80]. Interestingly, decreased JH is sufficient to extend lifespan in both sexes [81].

## Accumulation of endogenous toxins: excretory failure

### Excretory system

Despite physiological differences, *Drosophila* has an excretory system performing similar functions to the mammalian kidney. Flies have two pairs of Malpighian tubules that drain into the hindgut and onwards to the rectal ampulla. The tubules are important for eliminating toxic metabolic end-products, as well as regulating water and ionic balances [82].

Tubule secretion and junctional stability decrease with age [83]. Whether maintenance of these functions extends fly longevity remains unclear, but tubule dysfunction has been related to early mortality. For instance, flies on imbalanced high-caloric diets display dysregulation of purine catabolism and develop concretions in the excretory system [45, 84]. Tubule stones are associated with decreased secretion rates and shortened lifespan [45, 85].

Excretory failure may be a primary cause of death in the flies. Diet seems to be an important factor that can overwhelm the capacity of the excretory system to eliminate metabolic products, causing early death either directly by accumulating toxic metabolites or indirectly by blocking the system and dysregulating water and chemical balances.

## Accumulation of exogenous toxins: immunological decline

### Immunological decline

The fly immune system is mainly composed of haemocytes, macrophage-like circulating cells involved in processes such as wound healing and pathogen phagocytosis, and is comparable in function to mammalian innate immunity. In the absence of human intervention, infections are the principal cause of death of animals in the wild. Under lab conditions, excluding contamination with pathogenic organisms, infections in flies should only occur if commensal bacteria become pathogenic, either when the gut barrier integrity is compromised (see ‘Loss of intestinal homeostasis’) or by an impaired immune system with age [86].

ISC migration is induced as a protective mechanism after infection and epithelial injury. Due to ISC defects with age, this process is potentially impaired with ageing, which can sensitise flies to infection [87]. Although altered commensal microbiota seems to contribute to age-related changes in the gut [7, 88], the impact of microbiota on lifespan is inconsistent across studies and seems to be diet-dependent [89].

Fly innate immunity becomes impaired during ageing—the phagocytic ability of haemocytes declines with age [90, 91], and the number of haemocytes circulating in the haemolymph also decreases, although only in females [91]. Increased susceptibility to infection with age is seen in both sexes, but immune function is sexually dimorphic [92, 93], and thus may impact survival in a sex-specific manner.

Despite being dysfunctional, the immune response becomes hyperactive with ageing [94, 95]. Chronic inflammation predisposes flies to neurodegeneration and shortens their lifespan [95]. Increases in specific immune factors with age, such as antimicrobial peptides [96], may also be adaptive. Reduced activity of immunity pathways and delayed loss of intestinal barrier function, following overexpression of antimicrobial peptides, lead to infection resistance and lifespan extension in female flies [97].

## Loss of fluid homeostasis and compartmentalisation

### Water imbalance

Flies are particularly vulnerable to desiccation stress, surviving less than a day when deprived of water [98, 99]. This may be related to losses through their open respiratory system (see ‘Defective tracheal system’), although increasing environmental humidity partially prevents water loss but without impacting lifespan [100]. Cuticular hydrocarbons also play an important role in waterproofing the exoskeleton and protecting flies against fluid imbalance, from both environmental and nutritional stress, and can modulate survival [101].

Aged flies are more vulnerable to water imbalance, as the total volume of extracellular fluid decreases with age [45, 100]. Further dehydration stress caused by chronic consumption of high-sugar diets, for example, shortens fly survival, which can be fully rescued by water supplementation [45].

Loss of water can be a prevalent cause of death in a fly population if the food creates a chronic imbalance in osmolarity. Even under ideal dietary conditions, the aged excretory system can dysregulate fluid homeostasis not only by loss of water balance but also impaired excretion of key factors for osmolarity, such as sodium, glucose and urea. Additionally, changes in cuticle permeability related to differences in cuticular hydrocarbon composition and late life behavioural defects (i.e. loss of appetite or responsiveness to thirst signals) can also lead to desiccation [98, 102].

Besides water, fundamental chemical balances within fluids, such as acid base or redox homeostasis [103], may also lead to systemic failure and death if acutely disrupted or chronically impaired.

## Perspective

*Drosophila* ageing researchers should be encouraged to consider fly cause of death (Table 1). Tissue-targeted interventions in young adults that mimic altered function with age may provide insight into which pathophysiological processes are sufficient to cause systemic failure and how, if linked with broad functional assays and rescue experiments. Technological advances and increased availability of specialised approaches, such as ethoscopic monitoring [104] or micro-computed tomography imaging [105], may allow more advanced behavioural and morphological phenotyping, improving our ability to understand age-related fly pathologies and the pathophysiological changes that precede death. The establishment of a comprehensive functional scoring system in *Drosophila* for ageing studies, analogous to a human/mammalian frailty index, would help distinguish whether an intervention modulates fly longevity by acting on systemic ageing or by ameliorating a survival-limiting process in that specific population.

**Table 1:**
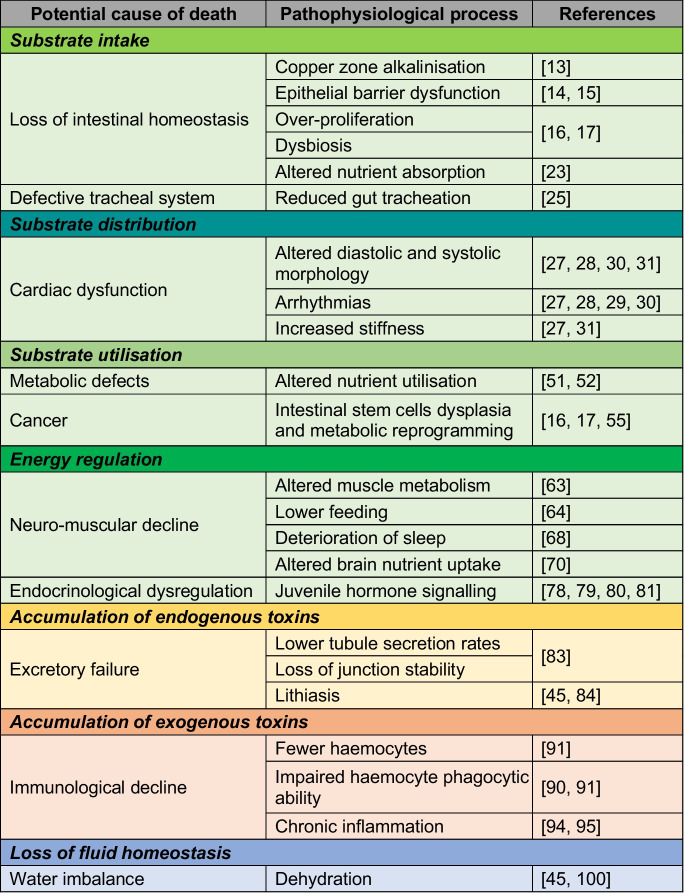
Summary of the different potential causes of death in *Drosophila*. Colour-coding corresponds to the scheme in Fig. 1

Different populations (i.e. genetic background, sex, culture conditions) are likely to have cohort-specific prevalence of diseases that cause death. Flies raised under a specific condition, that leads to the development of a lifespan-limiting pathology described above, will have a higher prevalence of death due to that process. Interventions that ameliorate this process will extend survival of that population, but may not be translatable to other populations that do not suffer from this same defect. Instead, ageing interventions should be translatable to all populations, since ageing is a risk factor that affects nearly all pathophysiological processes impacting survival (not just one). Therefore, multiple populations should be used to validate the effect of an intervention on lifespan.

The challenges of distinguishing ageing from survival also apply to mammalian model organisms, despite the ability to examine the animals *post mortem*. Given the time, cost and ethics of survival experiments, the scaling needed to apply an intervention to multiple populations is only feasible using short-lived species. *Drosophila* is a well-studied model organism from which fundamental biology applicable to humans has been discovered. Similarly, although ageing manifests differently in different species, its principles can be studied in the flies to ultimately find strategies to prevent age-related human frailty and maintain health and fitness for longer.
